# Severity of mitral valve prolapse is associated with basal left ventricular hypertrophy: a cardiac magnetic resonance study

**DOI:** 10.1186/1532-429X-14-S1-P99

**Published:** 2012-02-01

**Authors:** Mohammad I Zia, Valentina Valenti, Caroline Cherston, Maressa C Criscito, Seth Uretsky, Steven D Wolff

**Affiliations:** 1 Advanced Cardiovascular Imaging, Columbia University, New York, NY, USA; 2 Cardiology, Sunnybrook Health Sciences Centre, University of Toronto, Toronto, ON, Canada; 3 Medicine, St. Luke’s-Roosevelt Hospital Center, New York, NY, USA

## Summary

Mitral valve prolapse (MVP) is associated with concentric basal hypertrophy of the left ventricle. We found a strong correlation between the excursion of the mitral valve annulus and the degree of relative hypertrophy suggesting the possibility that locally increased myocardial function may be responsible for the hypertrophy.

## Background

**Figure 1:**
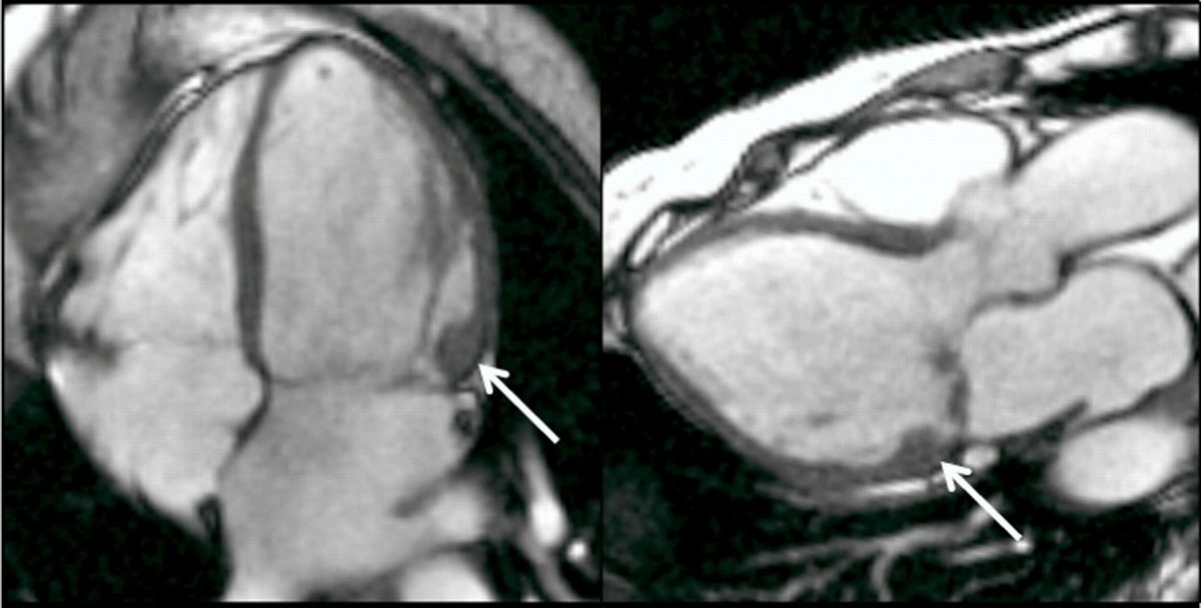
Focal basal lateral wall hypertrophy in a patient with mitral valve prolapse.

Recently, basal left ventricular hypertrophy has been suggested as a new form of hypertrophic cardiomyopathy. However, we have noticed that substantial focal basal hypertrophy often occurs in patients with mitral valve prolapse (Figure 1).

Our objective was to characterize the extent and distribution of focal basal left ventricular hypertrophy in patients with MVP and assess the correlation between the degree of focal hypertrophy and various myocardial structural parameters.

## Methods

Sixty-two patients (mean age: 58 years +/- 14; 56% males) with MVP and 20 age-matched normal volunteers (mean age: 53 years +/- 11; 50% males) were assessed using cardiac magnetic resonance imaging. We compared the ratio of basal to mid end-diastolic wall thickness in both groups and correlated it with various parameters such as age, left ventricular dimensions, degree of prolapse, mitral regurgitation volume, and mitral annular excursion.

## Results

**Figure 2:**
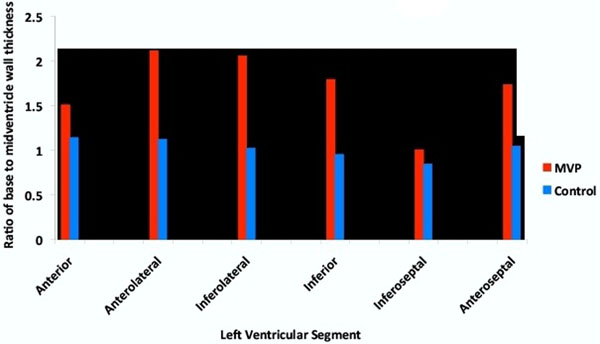
Relative basal left ventricular hypertrophy in patients with mitral valve prolapse.

Fourty-three (70%) patients had posterior leaflet prolapse, 2 (3%) patients had anterior leaflet prolapse and 17 (27%) patients had bileaflet prolapse. There was a significantly increased ratio of basal to mid end-diastolic wall thickness in all segments of the left ventricle in MVP patients when compared to controls (Figure 2). The inferolateral (2.1 vs. 1.0, p<0.01) and anterolateral ratios (2.1 vs. 1.1, p<0.01) were the highest compared to the other myocardial segments. Similar results were found when we analyzed the bileaflet and posterior leaflet prolapse patients separately. The degree of mitral annular excursion had a moderate linear correlation with the degree of hypertrophy (r2=0.72). However, age (r2=0.01), left ventricular end diastolic volume index (r2=0.01), left ventricular end systolic volume index (r2=0.01), degree of prolapse (r2=0.05), and mitral regurgitation volume (r2=0.02) did not have any significant correlation with the degree of hypertrophy.

## Conclusions

MVP is associated with concentric basal hypertrophy of the left ventricle. A moderate correlation between the excursion of the mitral valve annulus and the degree of relative hypertrophy suggests the possibility that locally increased myocardial function may be responsible for the hypertrophy. It is important to consider this mechanism when evaluating patients with basal hypertrophy to exclude the incorrect diagnosis of hypertrophic cardiomyopathy.

## Funding

None.

